# Characterization of epitranscriptome reader proteins experimentally and *in silico*: Current knowledge and future perspectives beyond the YTH domain

**DOI:** 10.1016/j.csbj.2023.06.018

**Published:** 2023-06-30

**Authors:** Lucas G. Miller, Madeline Demny, Phanourios Tamamis, Lydia M. Contreras

**Affiliations:** a McKetta Department of Chemical Engineering, The University of Texas at Austin, Austin, TX, USA; b Artie McFerrin Department of Chemical Engineering, Texas A&M University, College Station, TX, USA; c Department of Materials Science & Engineering, Texas A&M University, College Station, TX, USA; d Institute for Cellular and Molecular Biology, The University of Texas at Austin, Austin, TX, USA

**Keywords:** Epitranscriptomics, RNA binding proteins, YT-521B Homology (YTH) protein family, Molecular dynamics (MD) simulations, AlphaFold, Protein Structure Database

## Abstract

To date, over 150 chemical modifications to the four canonical RNA bases have been discovered, known collectively as the epitranscriptome. Many of these modifications have been implicated in a variety of cellular processes and disease states. Additional work has been done to identify proteins known as “readers” that selectively interact with RNAs that contain specific chemical modifications. Protein interactomes with N6-methyladenosine (m^6^A), N1-methyladenosine (m^1^A), N5-methylcytosine (m^5^C), and 8-oxo-7,8-dihydroguanosine (8-oxoG) have been determined, mainly through experimental advances in proteomics techniques. However, relatively few proteins have been confirmed to bind directly to RNA containing these modifications. Furthermore, for many of these protein readers, the exact binding mechanisms as well as the exclusivity for recognition of modified RNA species remain elusive, leading to questions regarding their roles within different cellular processes. In the case of the YT-521B homology (YTH) family of proteins, both experimental and *in silico* techniques have been leveraged to provide valuable biophysical insights into the mechanisms of m^6^A recognition at atomic resolution. To date, the YTH family is one of the best characterized classes of readers. Here, we review current knowledge about epitranscriptome recognition of the YTH domain proteins from previously published experimental and computational studies. We additionally outline knowledge gaps for proteins beyond the well-studied human YTH domains and the current *in silico* techniques and resources that can enable investigation of protein interactions with modified RNA outside of the YTH-m^6^A context.

## Graphical Abstract

**Figure:**
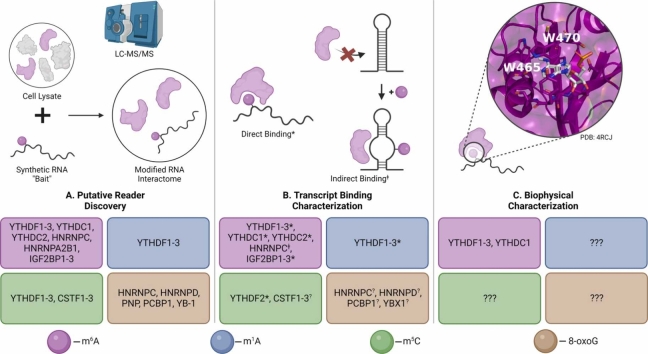

## 1 Introduction

Since the initial discovery of chemical modifications to RNA in the form of pseudouridine in 1957 [1], [2], published work has catalogued upwards of 150 modified versions of the four canonical RNA bases that make up what is known as the epitranscriptome; these modifications can be found across all domains of life and many types of RNA [3]. Modified bases have been mostly detected using several techniques that include separation and analysis by RNA chromatography and mass spectrometry methods [4]. More recently, next-generation sequencing has also been used to identify RNA modifications due to differences in their chemical properties from their unmodified base equivalents [3], [5]. Modified RNA species have been found in high abundance in transfer RNA (tRNA) and ribosomal RNA (rRNA) but can also be found in messenger RNA (mRNA) and other long non-coding RNAs (lncRNAs) [3]. The most abundant modified RNA base in eukaryotic mRNA identified thus far, N6-methyladenosine (m^6^A), has provided a wealth of insight into how RNA modifications might accumulate on transcripts and how their presence impacts cellular processes. For instance, the m^6^A modification has been shown to affect processes like alternative splicing of pre-mRNAs [5], [6], [7], cell growth and differentiation [8], [9], [10], and RNA localization [11].

**Fig. 1:**
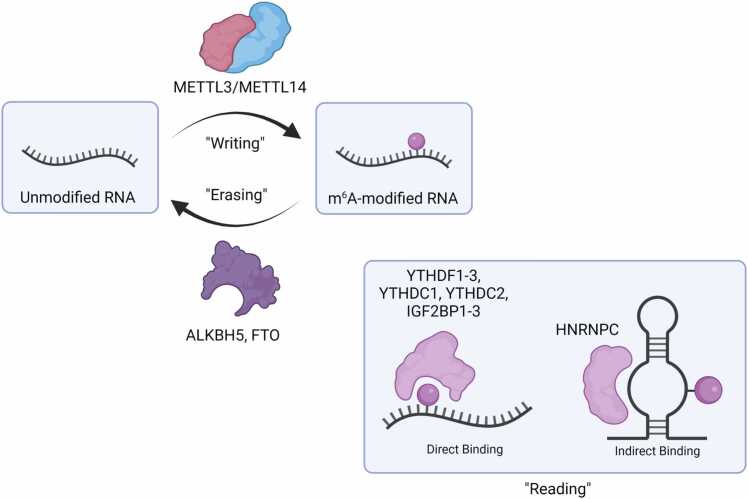
**Classes of proteins that regulate N6-methyladenosine (m**^**6**^**A).** The METTL3/METTL14 “writer proteins” acts to deposit a methyl group onto its RNA substrate to form an m^6^A-modified RNA [12]. This modification can be removed through the oxidative demethylation process catalyzed by the ALKBH family of proteins, including ALKBH5 and FTO [13], [14]. The m^6^A-modified RNA can be recognized by different “reader proteins” such as the YTH domain family and the IGF2BP family (IGF2BP1–3) through direct interactions with the RNA modification [9], [17]. Additionally, m^6^A can affect the local RNA structure of its transcript, allowing for indirect binding interactions with proteins such as HNRNPC [18]. Created with BioRender.com.

Characterization of the enzymes that deposit or remove the m^6^A mark has led to a more generalized model for dynamic “writing” or “erasing” of modifications to RNA (Fig. 1). In the case of m^6^A, enzymes known as “writer” proteins (*e.g.*, the METTL3/METTL14 complex [12]) add a methyl group to adenine to form m^6^A; removal of this chemical adduct is accomplished *via* enzymes referred to as “eraser” proteins (*e.g.*, FTO [13]) [14]. These proteins act to strike a cellular balance of m^6^A levels which, when disturbed, have implications in cancer proliferation and dysregulation of cellular processes [15]. In addition to the writers and erasers, the m^6^A mark is recognized by a class of proteins known as “readers” that bind to RNA containing this modification. The reader proteins for m^6^A selectively bind to modified transcripts over unmodified ones through either direct binding to the modified base and the bases flanking it, as is the case for the YT521-B homology (YTH) domain proteins [16] and the IGF2 binding proteins [17], or through indirect interactions with regions adjacent to the modified site, particularly in structured RNA regions, as with the HNRNPC protein [18]. These interactions between reader proteins and modified RNA can direct different transcripts for processes such as translation initiation, enhanced degradation, or for RNA localization [11], [19], [20], [21], [22], [23]. Other modifications are regulated by similar classes of proteins such as the ADAR1 writer protein for inosine [24], [25], [26] and the ALKBH eraser protein family for RNA methylations such as N1-methyladenosine (m^1^A), N3-methylcytosine (m^3^C), and m^6^A [27]. Although overall discovery and characterization of the writer and eraser proteins is important for understanding the regulatory networks of these modified RNAs (reviewed in [13], [27]), this minireview focuses on the reader proteins of m^6^A, m^1^A, N5-methylcytosine (m^5^C), and 8-oxo-7,8-dihydroguanosine (8-oxoG) [28], [29], [30]. Additionally, we acknowledge the numerous works to characterize the RNA sequence specificity of these protein readers [16], [31], [32], [33], [34] but limit our discussion to the biophysical investigations of protein-RNA interactions specific to modified RNA. To that end, we showcase the mechanistic understanding of protein interactions with the epitranscriptome that has been generated using both experimental and computational techniques. We discuss the power of the investigations that characterize the interactions between the YTH domain proteins and m^6^A, but we also outline current gaps in knowledge around the binding mechanisms for non-YTH readers of m^6^A and readers for other RNA modifications. Finally, we discuss the challenges and promise of conducting *in silico* biophysical studies of protein interactions with modified RNA and highlight current work in characterizing these interactions.

## 2 Characterization of the YT-521B homology (YTH) family using *in vitro* and *in silico* techniques

### 2.1 Discovery of the YTH protein family as m^6^A readers

Initial identification of proteins that recognize modified RNA transcripts has been accomplished through large scale RNA affinity chromatography studies [29], [30], [35], [36], [37]. In methods of this kind, a modified transcript acts as a “bait” for proteins that bind directly and indirectly (*i.e*., through protein-protein interactions or possibly RNA structure-specific interactions) to the modified RNA. These proteins are then isolated from whole cell lysate through affinity pulldown of the RNA [35], and mass spectrometry is used to determine the identity of the associated proteins. One such study [37] identified two proteins, YTHDF2 and YTHDF3, that interacted preferentially with an m^6^A-containing RNA bait *versus* an unmodified control. This was the first study to identify the YTHDF2 and YTHDF3 proteins as m^6^A interactors. In the case of YTHDF2 and YTHDF3, both proteins contain a YTH domain that is widely conserved across eukaryotes [31], [38]*.* After the discovery of YTHDF2 and YTHDF3 as putative m^6^A readers, these proteins as well as the three others found in humans that contain the YTH domain (YTHDF1, YTHDC1, and YTHDC2) were investigated for their recognition of m^6^A-containing transcripts *in vitro* [21], [28], [39], [40], [41], [42], [43], [44], [45]. Amino acid sequences corresponding to either the full YTH protein of interest or the isolated YTH domains were expressed recombinantly in *E. coli*, purified, and subjected to *in vitro* binding experiments (Table 1, [21], [28], [39], [40], [42], [43], [44], [45], [46]). These binding experiments, whose base principles are detailed in [47], [48], [49], [50], allowed for the calculation of protein affinity for a variety of RNA substrates *via* the dissociation constant (K_D_). It is difficult to directly compare the reported K_D_ values in some cases because of the effects of different buffer compositions, incubation temperatures, and incubation times on these values [51], [52]. However, common observations emerge from these studies regarding RNA containing m^6^A or the unmodified adenine. Regardless of RNA length, the YTH domains showed micromolar to sub-micromolar affinities and selectivity for m^6^A with respect to adenine. Specifically, these proteins showed either no binding to unmodified RNA [28], [45] or at least an order of magnitude difference in the K_D_ value to unmodified RNA for those studies that did show binding [21], [28], [40], [46]. An exception to this selectivity difference can be found in the electrophoretic mobility shift assays (EMSAs) for YTHDC2 [43] which showed a roughly two-fold increase in affinity for m^6^A-containing RNA, relative to unmodified RNA. Another common observation regarding m^6^A binding can be found in the effects of RNA sequence and length on K_D_. The YTH family of proteins has been shown to recognize predominantly a RRACH sequence motif, where R represents a purine base (A or G), A is modified to m^6^A, and H represents either A, C, or U [9], [53], [54] Alterations of this sequence motif, particularly in the position preceding the m^6^A modification (“G-1”), show the preference of YTHDC1 for RGAC [39], [42], [45]. Mutation of the “G-1” position to A lead to an increase in K_D_ from 0.3 μM to 2.0 μM for YTHDC1 [39], whereas the same RNA mutation shows only an increase of 1.0–1.1 μM for binding assays with YTHDF1. Additionally, 5-mer m^6^A-containing RNA sequences bound at lower levels of affinity to YTHDF1 and YTHDC1, which could be linked to the importance of regions flanking m^6^A for stabilizing the protein-RNA complex [42], [45]. Although these binding experiments with the wild-type protein sequences demonstrate the selectivity of the human YTH proteins and–more specifically–their domains for m^6^A, the key regions responsible for this selectivity needed to be determined. To elucidate the protein features responsible for the selective recognition of m^6^A containing transcripts, crystal structures were resolved in tandem with binding characterization efforts for all human YTH domains [39], [40], [41], [42], [43], [45], [55], [56], [57], [58], [59], [60].

**Table 1:** *In vitro* binding affinities for the human YTH domain family of proteins.

Protein	Domain/Full Protein	Technique	Incubation conditions	Oligomer Sequence (5’ - 3’)	K_D_ (nM)	Reference
YTHDF2	Full Protein	EMSA	Ice, 30 min	AUGGGCCGUUCAUCUGCUAAAAGG{m^6^A}CUGCUUUUGGGGCUUGU	179 ± 47	[21]
YTHDF2	Full Protein	EMSA	Ice, 30 min	AUGGGCCGUUCAUCUGCUAAAAGGACUGCUUUUGGGGCUUGU	2844 ± 656	[21]
YTHDF2	Full Protein	EMSA	Ice, 30 min	AUGGGCCGUUCAUCUGCUAAAACU{m^6^A}CUGCUUUUGGGGCUUGU	520 ± 155	[21]
YTHDF2	Full Protein	EMSA	Ice, 30 min	AUGGGCCGUUCAUCUGCUAAAAGGACUGCUUUUGGGGCUUGU	5187 ± 1330	[21]
YTHDF3	Full Protein	EMSA	Ice, 30 min	AUGGGCCGUUCAUCUGCUAAAAGG{m^6^A}CUGCUUUUGGGGCUUGU	323 ± 119	[21]
YTHDF3	Full Protein	EMSA	Ice, 30 min	AUGGGCCGUUCAUCUGCUAAAAGGACUGCUUUUGGGGCUUGU	1673 ± 1149	[21]
YTHDF1	Full Protein	EMSA	Ice, 30 min	AUGGGCCGUUCAUCUGCUAAAAGG{m^6^A}CUGCUUUUGGGGCUUGU	255 ± 46	[21]
YTHDC1	Domain	EMSA	4 °C, 1 hr	biotin-CCGUUCCGCCC{m^6^A}GGCCGCGCCCAGCUGGAAUGCA	700 ± 100	[28]
YTHDC1	Domain	EMSA	4 °C, 1 hr	biotin-CCGUUCCGCCC{m^1^A}GGCCGCGCCCAGCUGGAAUGCA	(23.3 ± 2.1) • 10^3^	[28]
YTHDC1	Domain	EMSA	4 °C, 1 hr	biotin-CCGUUCCGCCCAGGCCGCGCCCAGCUGGAAUGCA	(68.8 ± 13.9) • 10^3^	[28]
YTHDF1	Full protein	EMSA	4 °C, 1 hr	biotin-CCGUUCCGCCC{m^6^A}GGCCGCGCCCAGCUGGAAUGCA	1300 ± 100	[28]
YTHDF1	Full protein	EMSA	4 °C, 1 hr	biotin-CCGUUCCGCCC{m^1^A}GGCCGCGCCCAGCUGGAAUGCA	(16.5 ± 1.5) • 10^3^	[28]
YTHDF1	Full protein	EMSA	4 °C, 1 hr	biotin-CCGUUCCGCCCAGGCCGCGCCCAGCUGGAAUGCA	NB	[28]
YTHDF2	Full protein	EMSA	4 °C, 1 hr	biotin-CCGUUCCGCCC{m^6^A}GGCCGCGCCCAGCUGGAAUGCA	1300 ± 100	[28]
YTHDF2	Full protein	EMSA	4 °C, 1 hr	biotin-CCGUUCCGCCC{m^1^A}GGCCGCGCCCAGCUGGAAUGCA	5800 ± 1700	[28]
YTHDF2	Full protein	EMSA	4 °C, 1 hr	biotin-CCGUUCCGCCCAGGCCGCGCCCAGCUGGAAUGCA	NB	[28]
YTHDF3	Full protein	EMSA	4 °C, 1 hr	biotin-CCGUUCCGCCC{m^6^A}GGCCGCGCCCAGCUGGAAUGCA	1900 ± 100	[28]
YTHDF3	Full protein	EMSA	4 °C, 1 hr	biotin-CCGUUCCGCCC{m^1^A}GGCCGCGCCCAGCUGGAAUGCA	7000 ± 1000	[28]
YTHDF3	Full protein	EMSA	4 °C, 1 hr	biotin-CCGUUCCGCCCAGGCCGCGCCCAGCUGGAAUGCA	NB	[28]
YTHDC1	Domain	ITC	25 °C	GAACCGA{m^6^A}CUGUCUUA	2000 ± 400	[39]
YTHDC1	Domain	ITC	25 °C	GAACCGG{m^6^A}CUGUCUUA	300 ± 60	[39]
YTHDC1	Domain	ITC	25 °C	GAACCGC{m^6^A}CUGUCUUA	500 ± 120	[39]
YTHDC1	Domain	ITC	25 °C	GAACCGU{m^6^A}CUGUCUUA	400 ± 70	[39]
YTHDC1	Domain	ITC	25 °C	AAGAACCGG{m^6^A}CUGUCUUAGU	310 ± 70	[39]
YTHDC1	Domain	ITC	25 °C	AG{m^6^A}CU	3800 ± 400	[39]
YTHDC1	Domain	ITC	25 °C	GG{m^6^A}CU	2000 ± 100	[39]
YTHDC1	Domain	ITC	25 °C	UG{m^6^A}CU	4300 ± 500	[39]
YTHDF2	Domain	FP	4 °C, 30 min	FAM-UUCUUCUGUGGACUGUG	21.39 • 10^3^	[40]
YTHDF3	Domain	FP	4 °C, 30 min	FAM-UUCUUCUGUGG{m^6^A}CUGUG	2.54 • 10^3^	[40]
YTHDF1	Domain	ITC	25 °C	CCGA{m^6^A}CUGU	1100 ± 200	[45]
YTHDC1	Domain	ITC	25 °C	CCGA{m^6^A}CUGU	1000 ± 100	[45]
YTHDC1	Domain	ITC	25 °C	CCGG{m^6^A}CUGU	220 ± 30	[45]
YTHDC1	Domain	ITC	25 °C	CCGC{m^6^A}CUGU	320 ± 30	[45]
YTHDC1	Domain	ITC	25 °C	CCGU{m^6^A}CUGU	300 ± 60	[45]
YTHDF1	Domain	ITC	25 °C	CCGG{m^6^A}CUGU	800 ± 300	[45]
YTHDF1	Domain	ITC	25 °C	CCGC{m^6^A}CUGU	800 ± 200	[45]
YTHDF1	Domain	ITC	25 °C	CCGU{m^6^A}CUGU	900 ± 200	[45]
YTHDF1	Domain	ITC	25 °C	GAACCGA{m^6^A}CUGUCUUA	1100 ± 200	[45]
YTHDF1	Domain	ITC	25 °C	GAACCGG{m^6^A}CUGUCUUA	1000 ± 300	[45]
YTHDF1	Domain	ITC	25 °C	GAACCGC{m^6^A}CUGUCUUA	900 ± 200	[45]
YTHDF1	Domain	ITC	25 °C	GAACCGU{m^6^A}CUGUCUUA	1700 ± 400	[45]
YTHDF1	Domain	ITC	25 °C	AAGAACCGG{m^6^A}CUGUCUUAGU	1000 ± 100	[45]
YTHDF1	Domain	ITC	25 °C	AG{m^6^A}CU	(30 ± 4) • 10^3^	[45]
YTHDF1	Domain	ITC	25 °C	GG{m^6^A}CU	(22 ± 4) • 10^3^	[45]
YTHDF1	Domain	ITC	25 °C	UG{m^6^A}CU	(34 ± 4) • 10^3^	[45]
YTHDF1	Domain	ITC	25 °C	GAACCGGACUGUCUUA	NB	[45]
YTHDF1	Domain	ITC	25 °C	GGACU	NB	[45]
YTHDF2	Domain	ITC	25 °C	CCGA{m^6^A}CUGU	900 ± 200	[45]
YTHDF2	Domain	ITC	25 °C	CCGG{m^6^A}CUGU	900 ± 100	[45]
YTHDF2	Domain	ITC	25 °C	CCGC{m^6^A}CUGU	700 ± 200	[45]
YTHDF2	Domain	ITC	25 °C	CCGU{m^6^A}CUGU	800 ± 200	[45]
YTHDF2	Domain	ITC	25 °C	GAACCGA{m^6^A}CUGUCUUA	1500 ± 300	[45]
YTHDF2	Domain	ITC	25 °C	GAACCGG{m^6^A}CUGUCUUA	1700 ± 400	[45]
YTHDF2	Domain	ITC	25 °C	GAACCGC{m^6^A}CUGUCUUA	1300 ± 200	[45]
YTHDF2	Domain	ITC	25 °C	GAACCGU{m^6^A}CUGUCUUA	2000 ± 600	[45]
YTHDF2	Domain	ITC	25 °C	GAACCGGACUGUCUUA	NB	[45]
YTHDF2	Domain	ITC	25 °C	GGACU	NB	[45]
YTHDC2	Full Protein	EMSA	Ice, 30 min	ACCGGACUGUUACCAACACCCACACCCC-FAM	859.3 ± 281.2	[43]
YTHDC2	Full Protein	EMSA	Ice, 30 min	ACCGG{m^6^A}CUGUUACCAACACCCACACCCC-FAM	321.6 ± 61.9	[43]
YTHDC1	Domain	MST	NR	CGCGG{m^6^A}CTCTG (DNA)	9 ± 1	[44]
YTHDC1	Domain	MST	NR	CGCGG{m^6^A}CUCUG (RNA)	50 ± 10	[44]
YTHDC1	Domain	ITC	25 °C	CGCGG{m^6^A}CTCTG (DNA)	10 ± 1	[44]
YTHDC1	Domain	ITC	25 °C	CGCGG{m^6^A}CUCUG (RNA)	50 ± 10	[44]
YTHDF2	Domain	ITC	25 °C	CGCGG{m^6^A}CTCTG (DNA)	110 ± 10	[44]
YTHDF2	Domain	ITC	25 °C	CGCGG{m^6^A}CUCUG (RNA)	80 ± 20	[44]
YTHDF3	Domain	ITC	25 °C	CGCGG{m^6^A}CTCTG (DNA)	180 ± 20	[44]
YTHDF3	Domain	ITC	25 °C	CGCGG{m^6^A}CUCUG (RNA)	80 ± 10	[44]
YTHDF1	Domain	MST	25 °C, 2 hr	CGAGG{m^1^A}GGUGUAC-fluorescein	280 ± 60	[46]
YTHDF1	Domain	MST	25 °C, 2 hr	CGAGGAGGUGUAC-fluorescein	1250 ± 10	[46]
YTHDF2	Domain	MST	25 °C, 2 hr	CGAGG{m^1^A}GGUGUAC-fluorescein	620 ± 60	[46]
YTHDF2	Domain	MST	25 °C, 2 hr	CGAGGAGGUGUAC-fluorescein	2530 ± 20	[46]
YTHDF1	Domain	EMSA	4 °C, 1 hr	CGAGG{m^1^A}GGUGUAC-fluorescein	130 ± 47	[46]
YTHDF1	Domain	EMSA	4 °C, 1 hr	CGAGGAGGUGUAC-fluorescein	640 ± 200	[46]
YTHDF2	Domain	EMSA	4 °C, 1 hr	CGAGG{m^1^A}GGUGUAC-fluorescein	390 ± 30	[46]
YTHDF2	Domain	EMSA	4 °C, 1 hr	CGAGGAGGUGUAC-fluorescein	1380 ± 60	[46]
YTHDF1	Domain	EMSA	4 °C, 1 hr	CUUUU{m^1^A}AAGUAC-fluorescein	150 ± 44	[46]
YTHDF1	Domain	EMSA	4 °C, 1 hr	CUUUUAAAGUAC-fluorescein	770 ± 260	[46]
YTHDF2	Domain	EMSA	4 °C, 1 hr	CUUUU{m^1^A}AAGUAC-fluorescein	350 ± 48	[46]
YTHDF2	Domain	EMSA	4 °C, 1 hr	CUUUUAAAGUAC-fluorescein	≥ 1720 ± 530	[46]
YTHDC1	Domain	EMSA	4 °C, 1 hr	CGAGG{m^1^A}GGUGUAC-fluorescein	≥ 1857 ± 350	[46]
YTHDC1	Domain	EMSA	4 °C, 1 hr	CGAGGAGGUGUAC-fluorescein	≥ 361.1 ± 31	[46]
YTHDC1	Domain	EMSA	4 °C, 1 hr	CGAGG{m^6^A}GGUGUAC-fluorescein	68.4 ± 33	[46]
YTHDC1	Domain	EMSA	4 °C, 1 hr	CUUUU{m^1^A}AAGUAC-fluorescein	≥ 6185 ± 1180	[46]
YTHDC1	Domain	EMSA	4 °C, 1 hr	CUUUUAAAGUAC-fluorescein	≥ 2614 ± 2240	[46]
YTHDC1	Domain	EMSA	4 °C, 1 hr	CUUUU{m^6^A}AAGUAC-fluorescein	119.7 ± 10	[46]

Abbreviations: EMSA – Electrophoretic mobility shift assay; ITC – Isothermal titration calorimetry; FP – Fluorescence polarization; MST – Microscale thermophoresis; NR – Not reported in the referenced study; K_D_ – dissociation constant.

### 2.2 Crystallography studies identify similarities and differences in YTH domain structure

**Fig. 2:**
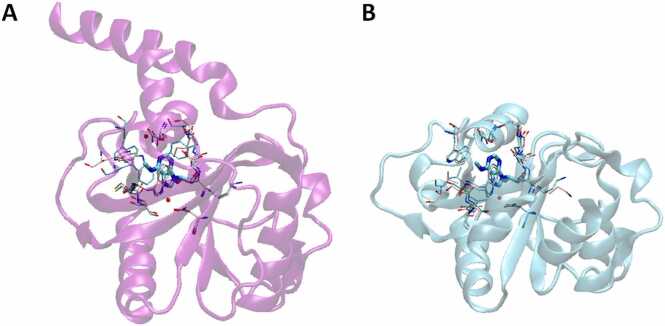
Experimentally resolved structures of human YTHDF1 and YTHDC1 domains determined by crystallography. Superposition was performed using iPBA web server [61]. In the descriptions below, all apo and holo structures have key interacting residues shown with pink and cyan carbon licorice, respectively. A) YTHDF1 holo structure bound to GG(m^6^A)CU 5mer RNA (PDB: 4rcj, YTHDF1 domain shown in purple cartoon) superimposed with YTHDF1 apo structure (PDB: 4rci). B) YTHDC1 holo structure bound to GG(m^6^A)CU 5mer RNA (PDB: 4r3i, YTHDC1 domain shown in cyan cartoon) superimposed with YTHDC1 apo structure (PDB: 4r3h).

**Fig. 3:**
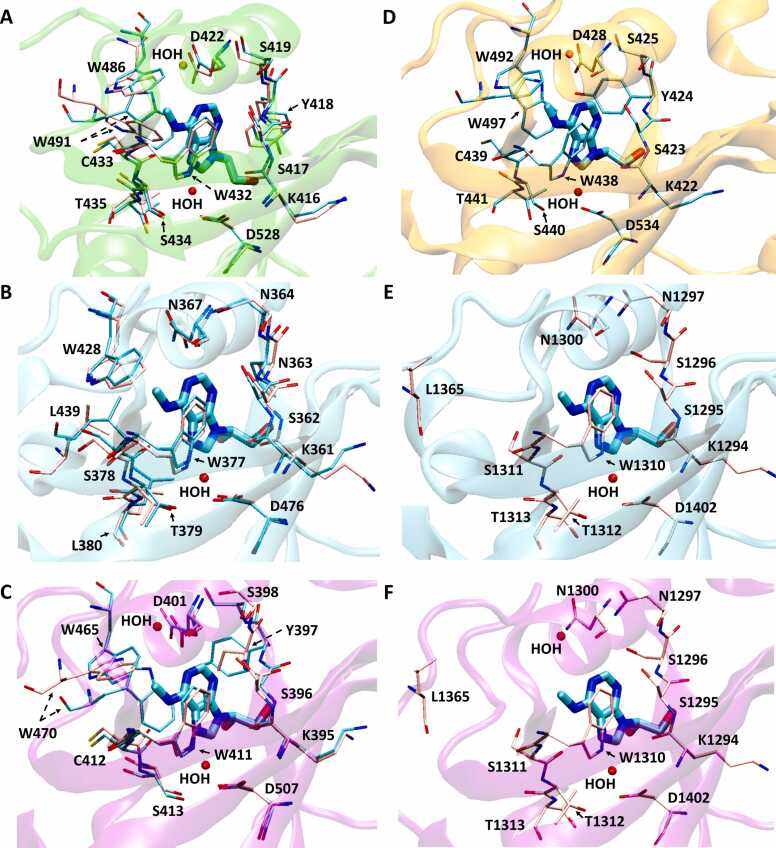
Experimentally resolved structures of human YTH domains determined by crystallography. Superposition was performed using iPBA web server [61]. In the descriptions below, all apo and holo structures have key interacting residues shown with pink and cyan carbon licorice, respectively. A) YTHDF2 apo structure (PDB: 4rdo) superimposed on YTHDF2 holo structure bound to mononucleotide m^6^A (PDB: 4rdn, YTHDF2 domain shown with green cartoon). B) YTHDC1 apo structure (PDB: 4r3h) superimposed on YTHDC1 holo structure bound to GG(m^6^A)CU 5mer RNA (PDB: 4r3i, YTHDC1 domain shown with cyan cartoon). C) YTHDF1 apo structure (PDB: 4rci) superimposed on YTHDF1 holo structure bound to GG(m^6^A)CU 5mer RNA (PDB: 4rcj, YTHDF1 domain shown with purple cartoon). D) YTHDF3 holo structure bound to GG(m^6^A)CU 5mer RNA (PDB: 6zot, YTHDF3 domain shown with orange cartoon). Apo structure not yet experimentally resolved. E) YTHDC2 apo structure (PDB: 6k6u) with m^6^A nucleotide and water molecule from YTHDC1 holo structure (PDB: 4r3i, m^6^A nucleotide shown with cyan carbon licorice, YTHDC1 domain shown with cyan cartoon). F) YTHDC2 apo structure (PDB: 6k6u) with m^6^A nucleotide and water molecules from YTHDF1 holo structure (PDB: 4rcj, m^6^A nucleotide shown with cyan carbon licorice, YTHDF1 domain shown with purple cartoon).

Studies have investigated human YTH domains in complex with m^6^A-containing RNA (referred to as bound or “holo” structures) and in the absence of RNA (referred to as unbound or “apo” structures) (Fig. 2, Fig. 3; superposition was performed with [61]). This investigation has enabled identification of similarities and differences of the binding mechanisms across YTH domains, as well as in the framework of apo *vs* holo states. The YTH domains share a hydrophobic pocket, as can be seen in the unbound (apo) and bound (holo) crystal structures of YTHDF2, that contains Tyr418, Trp432, Trp486, and Trp491 (Fig. 3 A) [40], [41], [60]. Two of these tryptophan residues, Trp432 and Trp486, were shown to be important for binding to m^6^A, as mutation of these residues to alanine markedly reduced affinity for an m^6^A-containing RNA [40]. The importance of this “aromatic cage,” a term coined by Xu and colleagues [39], in binding m^6^A-containing RNA was additionally depicted in the later crystallized YTH domain from YTHDF2 bound to a GG(m^6^A)CU pentanucleotide [41]. This binding pocket was also shown in the crystal structures of human YTHDC1 (Fig. 3 B) and later in those of YTHDF1 (Fig. 3 C), YTHDF3 (Fig. 3 D), and YTHDC2 (Fig. 3 E, 3 F) in both apo and m^6^A-bound (holo) forms [39], [45], [56], [59]. In each case, the aromatic cage was shown to be the site of m^6^A recognition. This was validated for YTHDC1 and YTHDF1 through mutagenesis of the corresponding tryptophan residues and subsequent *in vitro* binding assays [39], [45].

Although all the YTH domains from human proteins show similarity in their core aromatic binding cage for m^6^A recognition, the m^6^A-bound and unbound states show noteworthy differences. When the apo crystal structure of YTHDF2 was first resolved, the same study also resolved a holo crystal structure of YTHDF2 in complex with an m^6^A mononucleotide for comparison [60]. A local conformational adjustment of the loop between β4 and β5 (residues 476–492 in PDB: 4rdo) was observed in the presence of m^6^A. This loop contains Trp486 and Trp491, which accounts for two out of the four residues in the aromatic cage. Trp486 forms the “base” of the aromatic cage in both the apo and holo crystal structures of YTHDF2. The aromatic rings of Trp491, however, “flip” from the apo structure to become parallel to the aromatic rings of Trp432 in the holo structure [60]. These residues form the “walls” of the aromatic cage and the site of m^6^A recognition [60]. Similarly, in crystallography studies of YTHDC1, the loop between β4 and β5 (residues 418–440 in PDB 4r3h) was observed to be disordered and unresolved in the apo structure but resolved in the presence of m^6^A [39]. From the published crystal structures of YTHDC1, this sequentially homologous loop is also completely unresolved in apo structures of YTHDF1 and YTHDC2, including residues 460–469 [45] (PDB: 4rci) and residues 1354–1362 [56] (PDB: 6k6u), respectively. Overall, RNA binding induces some protein conformational stability to the loop. This trend holds for different human YTH domains [39], [41], [45], [56], [60], and certain residues share different orientations and positions in the bound *versus* unbound structures. These residues include Trp491 in YTHDF2, as well as Trp470 and Tyr397 in YTHDF1 (Fig. 3 A, 3 C). In YTHDF2, Tyr418 experiences a change in orientation compared to its homologues YTHDF1 and YTHDF3; this was attributed to the presence of a smaller ligand since YTHDF2 was crystallized with a m^6^A mononucleotide instead of a pentanucleotide (Fig. 3 A, 3 C, 3 D) [59]. Interestingly, in a more recent study [41], two orientations of Tyr418 were observed in the resolved YTHDF2 structure bound to a pentanucleotide; one of these orientations is similar to that depicted in Fig. 3 A while the other orientation is similar to that of its corresponding residues in YTHDF1 and YTHDF3 (Fig. 3 C, 3 D) [41]. Overall, the crystallography studies identified commonalities in the YTH domain binding pocket for m^6^A across these human proteins. Identification of the aromatic cage shed light on the mechanisms for m^6^A recognition, but the differences in conformational states in the unbound and bound forms of the protein, specifically in the homologous loop from YTHDC1 studies, highlighted the need for further study on the dynamics of the binding mechanisms for these proteins with m^6^A.

### 2.3 In silico investigation of the YTH domain from YTHDC1 identifies key conformational changes and solvent interactions responsible for the selectivity of m^6^A over adenine

To further investigate the interactions between the YTH domain and m^6^A, YTHDC1 was used as a model case for unbiased molecular dynamics (MD) simulations, alchemical transformations, and metadynamics [62]. Alchemical transformations to convert the N6 methyl group to an amino group were performed using the crystal structure of the YTH domain from YTHDC1 in complex with m^6^A to examine in detail the overall contribution of the methyl group to YTH domain binding [62]. In three steps, the partial charges were removed from the methyl group, converted to an uncharged amino group, and then recharged as an amino group to fully convert m^6^A to adenosine. The steps along this unphysical transformation were analyzed using thermodynamic integration (TI) [63] and showed that the methyl group represented a 16-fold difference in affinity with YTH between the m^6^A containing RNA and its unmodified counterpart [62]. Additionally, long unbiased MD simulations show that free GG(m^6^A)CU oligomer adopts a more favorable conformational state for association with the YTH domain in solvent relative to the GGACU unmodified RNA. When examining attributes of the binding pocket that could lead to m^6^A specificity, multiple microsecond-scale simulations of the apo YTH domain showed that conformational shifts of Met438 and Trp428 lead to metastable states that rearrange the structure of the aromatic binding pocket. In addition to the structural rearrangements, Thr379 was shown to be key for maintaining indirect interactions with m^6^A *via* a conserved water molecule. Follow-up crystallography experiments with a Thr379Val mutant to disrupt hydrogen bonding with the particular conserved water (referred to as “water 1”) resulted in a 140-fold decrease in affinity for a GG(m^6^A)CU RNA oligomer as measured by differential scanning fluorimetry [62]. The role of water solvation in the context of YTHDC1 was also investigated separately using unbiased simulations of the human YTH domain from YTHDC1 in the apo form and *Rattus norvegicus* YTH-G(m^6^A) bound form of this protein [64]. In the holo simulations, the N6 methyl group of m^6^A was shown to expel invading water molecules that occupied the m^6^A binding pocket in the apo simulations.

Water molecules involved in mediating interactions between YTH domain of YTHDC1 and m^6^A were resolved in the crystal structures of m^6^A-bound human YTH domains. In YTHDF1, a water molecule mediates interactions between the N7 of m^6^A and sidechains of Trp411 and Asp507 (Fig. 3 C). Similarly, a water molecule was resolved in the YTHDF2 bound structure to mediate interactions between the N7 of m^6^A and sidechains of Trp432 and Asp528 (Fig. 3 A) and in the YTHDF3 bound structure to mediate interactions with the sidechains of Trp438 and Asp534 (Fig. 3 D) [59]. The same water molecule described above plays a role in the binding of YTHDC1 to m^6^A by facilitating a hydrogen bond network between the N7 of m^6^A and Thr379 (Fig. 3 B) [62] This water molecule was also resolved in the apo structures of YTHDF1, YTHDF2, and YTHDC1 and mediates interactions between Trp411 and Asp507, Trp432 and Asp528, and Tyr379 in each of these domains, respectively [59], [62].

It is also important to note that another water molecule was resolved in the crystal structures for YTHDF2 and YTHDF1 in the bound but not unbound structure (Fig. 3 A, 3 C) [40], [45]. This water molecule was also resolved in the bound structure of YTHDF3 (Fig. 3 D), although an apo structure has not yet been experimentally resolved for a direct comparison [59]. In m^6^A-bound YTHDF1, the aromatic cage can be further stabilized through this water mediated interaction between Trp470 and Asp401. Similarly, this water molecule mediates the interaction between Trp491 and Asp422 in m^6^A-bound YTHDF2, where it has been observed that Trp491 changes orientation in the presence of m^6^A [40]. This water molecule was resolved in the m^6^A-bound YTHDF3 structure [59], mediating the interaction between Trp497 and Asp428 but is absent in the bound YTHDC1; interestingly, it was resolved in the unbound structure of YTHDC1, and was referred to as an unstable water molecule which is replaced upon RNA binding [62].

Taken together, the structural insights provided by these studies have allowed for a mechanistic understanding of m^6^A recognition through not only direct protein-RNA interactions, but also *via* protein interactions with solvent. This type of investigation of the YTH domain of YTHDC1 would be difficult with experimental techniques alone. These studies demonstrate the importance of combined computational and experimental approaches in uncovering how YTHDC1 interacts dynamically, and uniquely, with m^6^A-containing RNA through key structural rearrangements and the interaction with a mediating water molecule.

### 2.4 In silico investigation of YTH domain flexibility adds to the debate on the redundant function of the YTHDF1, YTHDF2, and YTHDF3 proteins

In addition to uncovering a mechanistic understanding of how the YTH domain from YTHDC1 recognizes m^6^A, *in silico* studies have also allowed for comparison of the binding pocket dynamics and recognition mechanisms for the other YTH domains. Although the YTH domains from human proteins have shown similar binding affinities for m^6^A containing RNA (Table 1), the similarity in function of these proteins *in vivo* is a topic of debate. While each protein possesses a YTH domain that shares high sequence and structural similarity with the other human YTH proteins, they have been suggested to actuate different functions *in vivo* [9]*.* YTHDF1 has been hypothesized to upregulate translation initiation of m^6^A-containing transcripts in HeLa cells [65]. This upregulation is thought to involve associations of the 40 S ribosomal subunit and translation initiation factors such as eIF3 with the N-terminal region of the protein. Similarly, YTHDF3 associates with the 40 S subunit but does not directly associate with subunits of the eIF3 translation initiation complex to upregulate translation [22]. In contrast to YTHDF3 and YTHDF1, YTHDF2 has been shown to promote destabilization and degradation of RNA through direct interaction with m^6^A containing transcripts and recruitment of the CCR4-NOT complex *via* a deadenylation mechanism [66]. This recruitment has been shown to be dependent on interactions between the CNOT1 SH domain and the N-terminal region of YTHDF2, rather than the C-terminal YTH domain. Although the YTHDF1 and YTHDF3 proteins seem to perform different functions than YTHDF2, all three proteins overlap considerably in the specific transcripts they interact with [22], [67]; examination of the crystal structures of the YTH domains from human YTHDF1 [45] and YTHDF2 [60] also shows that the m^6^A-binding residues and adjacent residues are conserved in the sequence of the YTH domain of YTHDF3, suggesting that these proteins may function redundantly to regulate the fate of m^6^A containing transcripts [67]. To further investigate the possible similarities in the conformational dynamics of these proteins, the unbound structures of the YTH domains of YTHDF1, YTHDF2, and YTHDF3 were subjected to 5 μs of simulation to compare the flexibility in the domains, particularly in the “recognition loop” containing two of the tryptophan residues responsible for forming the aromatic cage that recognizes m^6^A [59]. Root mean square fluctuation (RMSF) of regions within this recognition loop were found to be correlated across all three protein domains, with differences between conformational motion of the proteins found far from the binding pocket, suggesting similar structural dynamics of the domains in the context of m^6^A recognition [59]. This observation, along with the similarity in the bound structure of YTHDF3 to YTHDF2 [60] and YTHDF1 [45] described in Section 2.2, lends support to the hypothesis that these proteins recognize their m^6^A substrates in a similar manner. This study [59] investigated these proteins only in the context of their YTH domains, in absence of their N-terminal disordered regions. Further investigation is needed to place this data in context with the dissimilar associations of other protein factors with the N-terminal regions of the human YTH protein family [22], [65], [66], [68].

The ongoing investigation of the five YTH human proteins, experimentally and computationally, has led to a wealth of understanding around their binding properties. Specifically, *in silico* studies of YTHDC1 [62], [64] as well as YTHDF1, YTHDF2, and YTHDF3 [59] have provided significant insights as to how these m^6^A readers mechanistically recognize their modified substrate. Notably, future work is needed to understand in more detail the recognition properties of bound YTHDC2 in comparison to other YTH domains, and to uncover how these mechanisms affect the functions of the human YTH family of proteins overall.

### 2.5 Identification of small molecule inhibitors for the YTH domain family of proteins elucidates the druggability of epitranscriptome binding proteins

The regulation of m^6^A-containing transcripts by the YTH domain family of proteins has been implicated in a large variety of disorders (reviewed in [10], [15]) Due to their implications in human disease, the inhibition of these proteins with their cognate modified RNA substrates has been selected as a druggable target [41], [69], [70], [71]. YTHDC1 was initially selected as a candidate for small molecule drug discovery [69]. Through a fragment-based drug design methodology (reviewed in [72]), 30 small molecule fragments that consisted of m^6^A nucleobase analogs, uracil scaffold molecules, and other bicyclic compounds were identified to interact with the YTH domain of YTHDC1 [69]. These fragments were identified from computational docking of libraries of small molecules and further validated for their binding affinity using homogeneous time-resolved fluorescence (HTRF) [73] and isothermal titration calorimetry. Additionally, crystal structures of the protein-fragment complexes were generated to examine the binding modes of the small molecules. Of these, four fragments were found to show binding affinities below 1 mM and ligand efficiencies ranging from 0.25 to 0.4 kcal mol^-1^ n_HA_^-1^ [69]. Importantly, all of these fragments form interactions with the tryptophan residues that make up the aromatic cage (Trp377 and Trp428) and with Ser378, suggesting a link between interaction with these regions and metrics amenable to future drug development. In addition to these fragments, 25 small molecules were later identified through a similar computational and experimental pipeline [70]. These molecules included m^6^A base analogs, molecules containing an N-methyl amide that interacts with the polar residues Asn367 and Ser378 in the binding pocket, molecules containing a morpholine group that disrupts the recognition loop, and uracil derivatives that displace the structural water found in the binding pocket. Of these four classes of small molecules, the m^6^A analog with an N-methyl amide (referred to in the study as compound 6) was identified as a promising candidate for further design due to its IC_50_ of 39 μM and its ligand efficiency of 0.6 kcal mol^-1^ n_HA_^-1^. Additionally, this finding further underlines the importance of interactions with polar residues in the binding pocket of YTHDC1 and motivates additional development of a small molecule inhibitor for YTHDC1.

The YTHDF2 and YTHDF1 proteins have also been selected as targets for small molecule drug design. In addition to crystallizing the first structure of the YTH domain of YTHDF2 in complex with a pentanucleotide (PDB: 7z26) [41], Nai and colleagues identified 17 fragments from a combination of the previous YTHDC1 studies [70] and structure-based design that inhibit m^6^A-YTHDF2 binding activity. It is important to note that due to the structural and sequence similarity of YTHDF2 to YTHDF1 and YTHDF3, these small molecules might act as general inhibitors for all of these proteins [41]. The small molecules tested include m^6^A nucleobase and uracil analogs; as well as pyrazolopyrimidine, triazine, and pyrimidine derivatives. These molecules were analyzed for their inhibitory effects with HTRF, and their interactions with the YTH domain from YTHDF2 were determined using X-ray crystallography. One of these molecules, 6-cyclopropyluracil (referred to as compound 11) represents a promising candidate for future drug development with an IC_50_ of 174 μM and a ligand efficiency of 0.47 kcal mol^-1^ n_HA_^-1^, owing its potency to the interaction of the cyclopropyl group with the aromatic cage of YTHDF2 [41]. Furthermore, this study was the first of its kind to identify small molecule scaffolds for further drug discovery in the context of this YTH domain. In addition to the compounds identified as potential inhibitors of YTHDF2, the small molecule ebselen has also been proposed as a small molecule inhibitor of YTHDF1 [71]. Through a high-throughput tryptophan fluorescence quenching assay, ebselen was shown to directly inhibit binding of RNA both through *in vitro* binding assays and through immunoprecipitations from PC-3 prostate cancer cells treated with a non-lethal concentration of ebselen for 24 h. Ebselen was found to bind covalently with Cys412 through selenium sulfide bonds or reversibly with the m^6^A binding pocket depending on the reducing or oxidizing nature of the binding pocket environment as determined by X-ray crystallography [71]. Furthermore, the ebselen scaffold was used to design additional compounds with similar inhibitory characteristics, further demonstrating the use of the compound for further design efforts. Overall, the budding space of small molecule drug design for YTH domains offers exciting opportunities to probe the interaction dynamics of these proteins, both in the context of basic understanding of epitranscriptome regulation and in the context of human disease.

## 3 Characterization of epitranscriptome reader proteins beyond the YTH-m^6^A paradigm

### 3.1 The binding mechanisms for non-YTH m^6^A readers remain elusive

RNA chromatography studies that identify m^6^A-interacting proteins have also uncovered readers outside of the YTH family such as the insulin-like growth factor 2 binding proteins (IG2BPs) [17] and the heterogeneous nuclear ribonucleoproteins (HNRNPs), such as HNRNPC [5] and HNRNPA2B1 [7], that bind selectively to m^6^A-containing transcripts. The IGF2 binding proteins (IGF2BP1/IGF2BP2/IGF2BP3) have been found to bind m^6^A-containing transcripts with a 3-to-4-fold higher affinity relative to unmethylated transcripts [17]. Additionally, the RNA binding sites of these proteins overlap with sites of m^6^A methylation in both single-stranded and structured, hairpin RNA. These proteins contain two RNA recognition motif (RRM) and four K-homology (KH) RNA binding domains. The KH3 and KH4 domains of these proteins were shown to be key for binding a single-stranded m^6^A oligomer through RNA pulldown experiments followed by Western blotting to visualize the protein-RNA complex [17]. The IGF2BPs represent a departure from the canonical m^6^A binding pocket found in the YTH domain; however, the specifics of the structural similarities (or differences) between the m^6^A binding pockets of these proteins remains unclear. Furthermore, these proteins are currently being investigated as druggable targets in the context of colorectal cancer proliferation [74], and additional work on their interactions with m^6^A could elucidate the impact of a small molecule inhibitor on the IGF2BP-m^6^A interaction.

In addition to readers like the YTH proteins and the IGFBPs that bind directly to the modified m^6^A base, a subclass of proteins recognizes m^6^A in a more indirect, structurally dependent manner [75]. HNRNPC is one such m^6^A reader that is thought to bind to methylated transcripts *via* an indirect mechanism involving RNA structure [5]. One of the common secondary structures of RNA is known as a hairpin or stem loop, in which a single stranded region (or multiple regions) is flanked by double-stranded RNA (dsRNA) regions. This type of RNA structure can be impacted by the presence of an m^6^A modification, creating a structure distinct from its unmodified counterpart, which is preferably recognized for binding by the HNRPNC reader protein. One such example of this “m^6^A switch” behavior within the local RNA structure is found in the human metastasis-associated lung adenocarcinoma transcript (MALAT1) [5]; here, a portion of MALAT1 forms a 30-nucleotide stem loop containing the GGACU m^6^A methylation motif in a dsRNA region. Upon methylation of the adenine in this motif, the base pairing of adenine to uracil in the dsRNA region within the hairpin loop is disrupted, leading to a partial opening of the stem loop. The now single-stranded region of the partially opened stem loop previously bound to the GGACU motif becomes accessible to proteins like HNRNPC, which has been well characterized for its binding to MALAT1 in an m^6^A-dependent manner [5], [18], [76]. The recognition of MALAT1 by HNRNPC was shown to be structurally dependent through a GG(A→U)CU mutation in the MALAT1 stem loop, which mimics the base pairing disruption associated with an m^6^A methylation event [76]. Overall, from the aforementioned studies, HNRNPC represents a unique case of m^6^A “reading” as it appears not to recognize the m^6^A modification through direct binding, but rather recognizes unmodified regions of RNA that are made accessible for binding through structural rearrangements dependent on the presence of m^6^A. While other HNRNPs have been identified as m^6^A interacting proteins [37], many have not been further characterized as specific readers. One of these proteins, HNRNPA2B1, which has shown direct binding to transcripts containing m^6^A [7]; yet in another study, HNRPA2B1 was shown to exhibit a 1.6, 1.7 and 11.5-fold decrease in affinity for m^6^A modified RNA when compared with relative to unmodified for a 5-mer- 8-mer and 10-mer, respectively [77]. All in all, the mechanisms of HNRNPA2B1 binding to m^6^A is still an eluding problem. Thus, additional studies are needed to explore the nature of the molecular recognition mechanism.

Overall, non-YTH readers for m^6^A have only been recently investigated in terms of their mechanisms of binding to methylated transcripts. Excitingly, however, these investigations have not only identified a possibly novel mechanism for direct recognition of m^6^A, but also a potentially indirect, structurally dependent mechanism that might extend to other m^6^A reader proteins (besides HNRNPC). Further work is needed to characterize the dynamics of these binding events as well as to uncover alternative binding surfaces for m^6^A beyond the YTH aromatic cage.

### 3.2 Identification of proteins interacting with other modifications beyond m^6^A leads to further avenues of investigation for epitranscriptome recognition

In addition to the work that has been performed to identify interacting proteins with m^6^A modified RNAs, similar mass spectrometry techniques have been used to identify proteins interacting with m^1^A [28], m^5^C [29], and 8-oxoG [30] (Table 2, [5], [7], [11], [17], [21], [28], [29], [30], [37], [39], [40], [45], [56], [58], [59], [62], [76], [77], [78], [79], [80], [81], [82], [83]). Stable isotope labeling by amino acid in cell culture (SILAC) [84] has been used to identify proteins interacting with m^1^A with a 34-mer RNA probe designed to carry a portion of the *SOX18* gene known to be modified *in vivo* [28], [36], [85]. From subsequent liquid chromatography tandem mass spectrometry (LC-MS/MS) analysis, the YTH domain family proteins YTHDF1, YTHDF2, and YTHDF3 as well as other proteins such as the heterogeneous nuclear ribonucleoprotein hnRNPD and the TAR DNA-binding protein were identified as putative m^1^A readers. Follow-up *in vitro* binding characterization was conducted with the YTH domain proteins *via* EMSA [28]. These assays showed that the YTHDF1, YTHDF2 and YTHDF3 proteins, as well as the YTH domain from YTHDC1, bound to the same SOX18 RNA oligomer containing m^1^A at a lower affinity relative to the same oligomer containing m^6^A. Despite this lower affinity, these proteins were selective for both m^1^A and m^6^A relative to an unmodified RNA oligomer containing adenine (Table 1). To determine if the binding mechanism of the YTHDF2 with m^1^A modified RNA is like the binding mechanism of these proteins with m^6^A modified RNA, mutagenesis was performed for the YTHDF2 protein followed by EMSAs to determine relative changes in affinity for the m^1^A-containing RNA substrate [28]. Specifically, mutation of one of the key tryptophan residues that make up the “aromatic cage” responsible for m^6^A modified RNA recognition (Trp432 in YTHDF2) led to an abrogation of m^1^A binding activity, suggesting that m^1^A modified RNA may be recognized through a similar mechanism to m^6^A modified RNA; however, the other proteins identified in the m^1^A interactome from this study were not investigated further. The large difference in affinity of these YTH proteins for m^1^A and m^6^A modified RNA was later investigated using follow-up EMSAs and microscale thermophoresis (MST) experiments [46]. This study observed sub-micromolar K_D_ values with m^1^A containing oligomers for both YTHDF1 and YTHDF2, as well as a 3-to-5-fold decrease in affinity for an equivalent unmodified oligomer (Table 1). Interestingly, this study also contradicts the initial claim that YTHDC1 recognizes m^1^A, showing no selectivity for m^1^A over unmodified RNA, suggesting that “m^1^A recognition is specific to YTHDF1/2” [46]. YTHDF2 also appears in the list of m^5^C associated proteins, along with the cleavage stimulation factor proteins CSTF1, CSTF2, and CSTF3 (Table 2). While these proteins were investigated for their direct binding to m^5^C, only YTHDF2 was selected for quantitative binding characterization *via* EMSA. This protein shows a higher binding affinity to m^5^C than unmodified RNA and shows binding dependence on the same Trp432 within its aromatic cage found to be relevant for m^1^A and m^6^A binding [29]. These results have suggested that the recognition capabilities of at least some of the YTH domain proteins could extend beyond m^6^A but also highlight the unexplored mechanisms of RNA modification recognition. Finally, 8-oxoG reader proteins such as HNRNPD, PCBP1, YB-1, and HNRNPC have been investigated *in vitro* to varying degrees with respect to their direct binding to modified RNA [30], [80], [81], [82]. HNRNPD has been shown to bind to 8-oxoG containing transcripts with high affinity through both RNA pulldown experiments and subsequent Western blotting [30], [81]. Interestingly, PCBP1 has also shown specific binding 8-oxoG over unmodified RNA but shows preference for two of these modified bases spaced 6 nucleotides apart rather than a single modification as shown for other proteins [82], showcasing the diversity in binding behavior of the studied 8-oxoG readers. The YB-1 protein has been shown to readily form complexes with RNA containing 8-oxoG [80], and the central protein region–along with the C-terminus–is required for modification binding. Interestingly, YB-1 has been shown to associate with the IGF2BPs to regulate MYC and BCL2 RNA transcripts in an m^6^A dependent manner [86] which could suggest interplay between the two modified RNA pools. However, the binding dynamics for YBX1 and 8-oxoG are not well understood, and more work is needed to understand the 8-oxoG recognition mechanism by these proteins both *in silico* and *in vitro*. No such investigation has been performed *in silico*, despite the availability of numerous crystal structures in complex with an RNA strand (Table 2). Similarly, although a crystal structure for HNRNPC has been resolved, its RRM binding domain has yet to be studied mechanistically for 8-oxoG recognition using *in vitro* or *in silico* techniques. In summary, the discovery of YTH proteins and others that recognize modifications beyond the well-studied m^6^A represent exciting opportunities to not only identify how reader proteins might flexibly recognize the epitranscriptome, but also to identify other characteristic mechanisms of chemically modified RNA recognition by reader proteins.

**Table 2:** Epitranscriptome-associated proteins with confirmed direct binding to modified RNA transcripts.

Gene Name	Associated Modification	Domains	Discovery method	Direct transcript binding shown?	Notes	Ref.	Relevant crystal structures (PDB)
HNRNPA2B1	N6-Methyladenosine	RRM_1 (1), RRM_6 (1)	RNA affinity pulldown, LC-MS/MS	HITS-CLIP, UV-CLIP, RNA protection assay, RNA pulldown, immunoblotting	Proposed to act in an "m^6^A switch" dependent mechanism, rather than direct binding[64]	[7], [37], [77]	5HO4
HNRNPC	N6-Methyladenosine	RRM_1 (1)	RNA affinity pulldown, LC-MS/MS	Filter-binding assay	associates with m^6^A-switch constructs by binding the U-tract formed in methylated hairpins	[5]	2MZ1
IGF2BP1	N6-Methyladenosine	RRM_1 (1), KH_1 (4), RRM_6 (1)	LC-MS/MS and computational prediction of m^6^A binding proteins	EMSA	Direct binding demonstrated *in vitro* and *in vivo,* KH3/4 indispensable in binding to m^6^A	[17]	6QEY, 2N8L
IGF2BP2	N6-Methyladenosine	RRM_1 (2), KH_1 (4)	LC-MS/MS and computational prediction of m^6^A binding proteins	EMSA	Direct binding demonstrated *in vitro* and *in vivo,* KH3/4 indispensable in binding to m^6^A	[17]	6ROL
IGF2BP3	N6-Methyladenosine	RRM_1 (1), KH_1 (4), RRM_6 (1)	LC-MS/MS and computational prediction of m^6^A binding proteins	EMSA	Direct binding demonstrated *in vitro* and *in vivo,* KH3/4 indispensable in binding to m^6^A	[11], [17]	6FQR
Prrc2a	N6-Methyladenosine	BAT (1)	RNA affinity pulldown, LC-MS	EMSA	direct binding demonstrated *in vitro*, associated with oligodendroglia proliferation	[79]	
RBMX (aka HNRNPG)	N6-Methyladenosine	RBM1CTR (1), RRM_1 (1)	RNA affinity pulldown, LC-MS/MS	EMSA	binds m^6^A through C-terminal low-complexity region	[76]	2MB0
YTHDC1	N6-Methyladenosine	YTH (1)	RNA affinity pulldown, LC-MS/MS	ITC	binds m^6^A using an "aromatic cage"	[37], [39], [45], [62]	6ZCN
YTHDC2	N6-Methyladenosine	OB_NTP_bind (1), Ank_2 (1), HA2 (1), Helicase_C (1), YTH (1), R3H (1), DEAD (1)	SILAC-based RNA pulldown, LC-MS/MS	ITC	shown to have a conserved m^6^A binding pocket and shares similarities to other YTH domains	[11], [45], [56]	6K6U
YTHDF1	N6-Methyladenosine	YTH (1)	RNA affinity pulldown, LC-MS/MS	EMSA, ITC	binds directly to m^6^A, forms conserved aromatic cage to recognize the modification	[21], [45]	4RCJ
YTHDF2	N6-Methyladenosine	YTH (1)	RNA affinity pulldown, LC-MS/MS	FP, EMSA, ITC	YTH domain binds directly to m^6^A and shares similarity in structure to YTHDC1; basic residues near the binding cage of YTH domain	[37], [40], [45]	4RDN
YTHDF3	N6-Methyladenosine	YTH (1)	RNA affinity pulldown, LC-MS/MS	EMSA	YTH domain binds directly to m^6^A. YTH domain selects for m^6^A containing RNA *via* loop-loop interactions, conformation selectivity, and induced fit effects.	[37], [58], [59]	6ZOT
ALYREF	N5-Methylcytosine	FoP_duplication (1), RRM_1 (1), FYTT (1)	RNA Immunoprecipitation MS/MS	EMSA	Lys171 key for recognition of m^5^C (found by comparing MBD and YTH domain sequences and performing point mutations)	[78]	1NO8
CSTF1	N5-Methylcytosine		SILAC-based RNA pulldown, LC-MS/MS	RNA pulldowns, Western blotting		[29]	
CSTF2	N5-Methylcytosine	RRM (1)	SILAC-based RNA pulldown, LC-MS/MS	RNA pulldowns, Western blotting		[29]	
CSTF3	N5-Methylcytosine		SILAC-based RNA pulldown, LC-MS/MS	RNA pulldowns, Western blotting		[29]	
YTHDF1	N5-Methylcytosine	YTH (1)	SILAC-based RNA pulldown, LC-MS/MS	RNA pulldowns, Western blotting		[29]	
YTHDF2	N5-Methylcytosine	YTH (1)	SILAC-based RNA pulldown, LC-MS/MS	RNA pulldowns, Western blotting, EMSA	Trp432Ala mutation leads to loss of m^6^A affinity	[29]	
YTHDF3	N5-Methylcytosine	YTH (1)	SILAC-based RNA pulldown, LC-MS/MS	RNA pulldowns, Western blotting		[29]	
YTHDF1	N1-Methyladenosine	YTH (1)	RNA affinity pulldown, LC-MS/MS	EMSA		[28]	4RCJ
YTHDF2	N1-Methyladenosine	YTH (1)	RNA affinity pulldown, LC-MS/MS	EMSA		[28]	4RDN
YTHDF3	N1-Methyladenosine	YTH (1)	RNA affinity pulldown, LC-MS/MS	EMSA		[28]	6ZOT
HNRNPC	8-oxo-7, 8-dihydroguanosine	RRM_1 (1)	RNA affinity pulldown, LC-MS/MS	RNA pulldowns, Western blotting, competition experiment		[30]	2MZ1
HNRNPD (AUF1)	8-oxo-7, 8-dihydroguanosine	RRM_1 (2), CBFNT (1)	RNA affinity pulldown, LC-MS/MS	RNA pulldowns, Western blotting		[30], [81]	5IM0, 1X0F
PCBP1	8-oxo-7, 8-dihydroguanosine	KH_1 (3)	RNA affinity chromatography coupled with mass spectrometry	RNA pulldowns, Western blotting	Binds to 2 8-oxoG residues with higher preference than a single mark (positions 9 and 15 in a 30mer RNA oligomer)	[82]	1ZTG, 1WVN
PNPT1	8-oxo-7, 8-dihydroguanosine	S1 (1), PNPase (1), RNase_PH (2), RNase_PH_C (2), KH_1 (1)	RNA protection assays followed by SDS-PAGE analysis	EMSA		[83]	4AM3
YBX1	8-oxo-7, 8-dihydroguanosine	S1 (1)	RNase A protection assay, gel shift assay (EMSA)	EMSA	Central and C-terminal protein regions required for 8-oxoG binding activity	[80]	5YTX, 5YTY, 5YTV, 5YTS

Abbreviations: Ref. – reference; LC-MS/MS – Liquid chromatography coupled with mass spectrometry; SDS-PAGE – Sodium dodecyl sulfate (denaturing) polyacrylamide gel electrophoresis; HITS-CLIP - High-throughput sequencing of RNA isolated by crosslinking immunoprecipitation; UV-CLIP – UV cross-linking and immunoprecipitation; EMSA – Electrophoretic mobility shift assay; ITC – Isothermal titration calorimetry; FP – Fluorescence polarization; MST – Microscale thermophoresis.

## 4 Computational advancements accelerating the study of epitranscriptome reader proteins *in silico*

Computational advancements have played a key role in the *in silico* investigation of protein-RNA interactions, including interactions with RNA modifications. In the context of computational studies, MD simulations can be considered a potent tool to study the structure and dynamics, and provide biophysical insights for such interactions starting from an initial protein-RNA structural conformation. The development of molecular mechanics force-fields, programs as well as platforms to build, simulate and analyze such systems has significantly enabled the computational study of RNA-protein interactions using MD simulations [87], [88], [89], [90], [91], [92], [93], [94]. If the structure under investigation has not been experimentally resolved, computational methods can also be used for its initial modeling. Computational methods, when combined with experimental techniques as shown above, represent powerful synergistic approaches for the identification and biophysical characterization of novel protein interactions with modified RNAs.

### 4.1 Computational methods for protein structure prediction

Historically, the investigation of protein-RNA interactions using MD simulations has been limited partly due to the lack of crystallography data for the protein of interest. The advent of structural modeling tools has allowed the generation of protein structures from a primary amino acid sequence, which can serve as a starting point for further modeling of protein-RNA interactions. One such method of structure prediction involves homology modeling and is leveraged by tools such as I-TASSER [95], Phyre2 [96], HHPred [97] and Modeller [98]. A homology modeling approach builds a protein structure based on fragments of experimentally resolved structures from the Protein Data Bank [99] that share homology with the input primary sequence [100]. These fragment “templates” are then threaded together to generate the structural model of the input protein, which is checked and further refined using a variety of energetic analyses and iteration of the structure building process [95]. For example, I-TASSER and Phyre2 have been used to identify biologically relevant structural and functional features of proteins. I-TASSER was used to predict the structure of caveolin-1 (cav-1), a membrane-associated protein, with HNRNPA2B1, an RNA binding protein responsible for binding microRNA (miRNAs) such as mi-R17/93 present in the resulting microvesicles, which package these miRNA species to control gene expression during periods of oxidative stress [101]. The structural model of the cav-1/HNRNPA2B1 complex correctly predicted the caveolin scaffolding domain (CSD) of cav-1 and the arginine-glycine-glycine (RGG) repeat box of HNRNPA2B1 to be key for complex formation. This predicted protein-protein interaction was confirmed *in vitro* through inhibition of HNRNPA2B1/cav-1 binding by competition with a CSD peptide and through RGG deletions from HNRNPA2B1 in immunoprecipitation experiments [101]. Another homology modeling tool, Phyre2, was used predict the structure of CcaF1, a previously uncharacterized protein in the archaea *Rhodobacter sphaeroides* [102]. The predicted structure of this protein showed homology to the RNA binding domain in the Smaug protein from *D. melanogaster*, suggesting that CcaF1 might be responsible for binding and regulation of RNA. This prediction was confirmed, as CcaF1 was shown to bind the small RNA CcsR1 *in vitro* and regulate its stability *in vivo* [102].

**Fig. 4:**
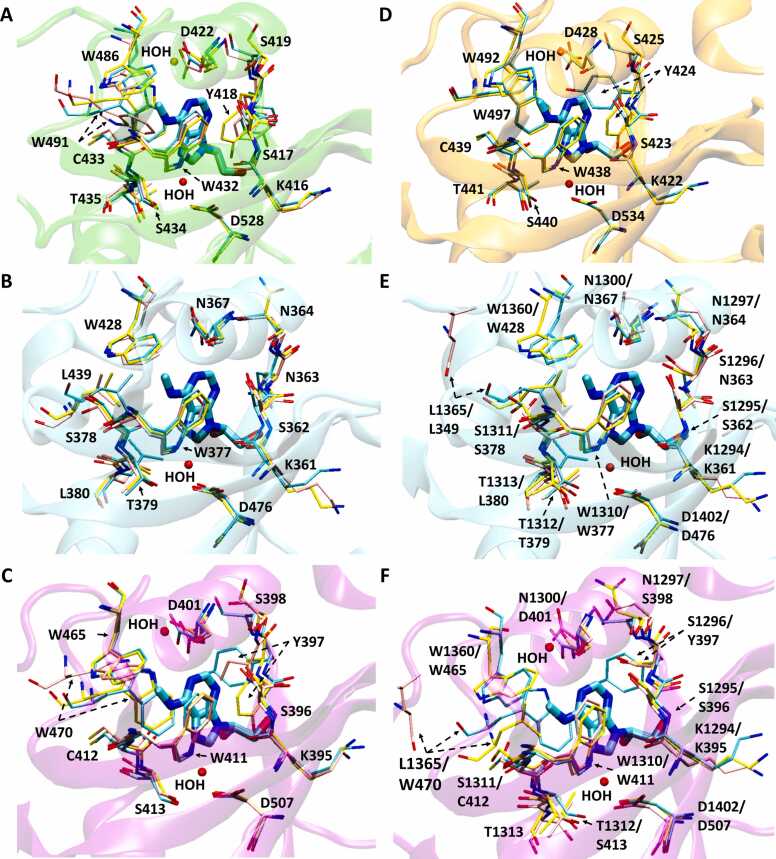
Experimentally resolved structures of human YTH domains determined by crystallography and their corresponding AlphaFold models. Superposition was performed using iPBA web server [61]. In the descriptions below, all apo, holo, and AlphaFold structures have key interacting residues shown with pink, cyan, and yellow carbon licorice, respectively. A) AlphaFold model of YTHDF2 (UniProt Q9Y5A9) superimposed on YTHDF2 apo structure (PDB: 4rdo) and YTHDF2 holo structure bound to mononucleotide m^6^A (PDB: 4rdn, YTHDF2 domain shown with green cartoon). B) AlphaFold model of YTHDC1 (UniProt Q96MU7) superimposed on YTHDC1 apo structure (PDB: 4r3h) and YTHDC1 holo structure bound to GG(m^6^A)CU 5mer RNA (PDB: 4r3i, YTHDC1 domain shown with cyan cartoon). C) AlphaFold model of YTHDF1 (UniProt Q9BYJ9) superimposed on YTHDF1 apo structure (PDB: 4rci) and YTHDF1 holo structure bound to GG(m^6^A)CU 5mer RNA (PDB: 4rcj, YTHDF1 domain shown with purple cartoon). D) AlphaFold model of YTHDF3 (UniProt Q7Z739) superimposed on YTHDF3 holo structure bound to GG(m^6^A)CU 5mer RNA (PDB: 6zot, YTHDF3 domain shown with orange cartoon). Apo structure not yet experimentally resolved. E) AlphaFold model of YTHDC2 (UniProt Q9H6S0) superimposed on YTHDC2 apo structure (PDB: 6k6u) and YTHDC1 holo structure bound to GG(m^6^A)CU 5mer RNA (PDB: 4r3i, YTHDC1 domain shown with cyan cartoon). F) AlphaFold model of YTHDC2 (UniProt Q9H6S0) superimposed on YTHDC2 apo structure (PDB: 6k6u) and YTHDF1 holo structure bound to GG(m^6^A)CU 5mer RNA (PDB: 4rcj, YTHDF1 domain shown with purple cartoon).

Most recently, the field of protein structure prediction was revolutionized by the deep-learning neural network-based method AlphaFold, which improves upon previous homology-based approaches (reviewed by [103]). It is worth noting that in the 2020 Critical Assessment of Structure Prediction (CASP14), AlphaFold demonstrated accuracy on par with experimentally resolved structures in a majority of cases and significantly outperformed other computational methods [104]. Additionally, AlphaFold demonstrated the capacity to predict the structure of many difficult protein targets at or near experimental resolution [105]. AlphaFold’s success could be attributed to certain key factors, including its methodology and the fact that the single domain protein structure library is basically complete [106]. The advancement of protein structure prediction methods is a key contributing factor to the study of protein interactions with molecules such as RNA, DNA, other proteins, and small molecules. Given that AlphaFold was applied to a wide range of proteins, a significant number of RNA binding protein structures that have not been resolved by experimental approaches have been predicted by AlphaFold [107]. As a result, AlphaFold could largely increase the overall capacity to study protein-RNA recognition, including protein interactions with modified RNAs, due to its ability to predict the overall protein structures with high-accuracy. While AlphaFold can predict a protein structure with accurate conformational packing of the backbone and side chains, the modeled structure could correspond to a particular conformation, as is the case for proteins that show different conformations when in active or inactive states [106]. A protein can exist in one conformation when it is in its bound state, and in another conformation when it is in its unbound state. Therefore, a careful consideration and inspection of AlphaFold predicted models over different known states could be worthy of investigation. In this context, we took the initiative to compare AlphaFold-predicted models provided within the AlphaFold Protein Structure Database [104], [108] for the case of YTH human domains with experimentally resolved bound and unbound structures (Fig. 4). The AlphaFold models predict backbone conformations accompanied by sidechain orientations that more closely resemble those of the experimental structures in the bound state for YTHDF2, YTHDC1, and YTHDF1 (Fig. 4 A, 4 B, 4 C, [39], [45], [59], [60]). Notably, one particular residue in the AlphaFold model of YTHDF1, Tyr397, has a different orientation than its holo structure (Fig. 4 C). This orientation is reminiscent of its corresponding residue Tyr418 in YTHDF2 holo structure in complex with a mononucleotide (PDB: 4rdn; Fig. 2 A) [60], and one of the two conformations in a more recently resolved YTHDF2 holo structure in complex with a pentanucleotide (PDB: 7z26) [41]. For the AlphaFold model of YTHDF3, which does not have an experimentally resolved apo structure for comparison, similarities are observed in the conformation of most of the aromatic side chains to the bound experimental structure (Fig. 4 D). Similar to the AlphaFold model of YTHDF1, Tyr424 in YTHDF3 adopts an orientation mimicking that of Tyr418 in the same relative position for experimentally resolved structures of YTHDF2 (Fig. 4 A, 4 D). Importantly, in the case of YTHDC2, for which a bound state is not experimentally available, the sidechain positions (especially of YTHDC2 residue Leu1365) of the AlphaFold model shows close resemblance to both bound YTHDF1 and YTHDC1 (Figs. 4 E, 4 F). AlphaFold models predict the YTH domains closer to the bound rather than unbound state, which can possibly be attributed to the fact that unbound YTH domains contain partially unresolved residue moieties; this could potentially be related to the fact that binding sites (such as the YTH RNA binding domain), are in general the most accurately predicted regions of a protein’s conformation [106].

### 4.2 Methods for generating a biomolecular interaction model

An “appropriate” protein structure (*i.e*., either experimentally resolved in the bound state or a computationally predicted structure) can serve as a stepping stone to study and identify the protein-RNA interface, and subsequently build an initial interaction model between protein and RNA [107]. This model can be generated using a variety of methods–analogous to methods developed from protein-protein interactions–including rigid-body docking [109], template-based docking [107], [109], [110], and other machine-learning based methods [110], [111], [112]. Rigid body docking, which searches and superimposes static input structures based on favorable energetics of the resulting complex, is useful for determining an initial interaction model for a protein-RNA complex; however, the highly flexible and dynamic nature of RNA molecules may lead to biased docking based on the input conformation [109]. In template-based (or comparative) docking, the structural similarity between the complex to be modeled and an experimentally resolved complex is assessed. This modeled complex can be constructed by superposition (*e.g.*, of the monomer models with respect to the experimentally resolved complex) and then evaluated through scoring functions measuring structural similarities between the monomer models, as well as the complex template components [109], [110]. In such comparative docking approaches, the choice of experimental template by a local alignment to the complex interfaces over alignment to the entire complex can slightly improve the quality of the modeled complex, as indicated for protein-protein interactions involving a binding-induced conformational change [113]. This approach could also hold when modeling interactions between proteins with nucleic acids as well, given their dynamic nature. However, a caveat to such alignment methods comes from what is known as the “twilight zone” (roughly 25%) of overall sequence similarity found when studying protein-protein interactions [109], [114]. Nevertheless, when similarity between the template and modeled complexes is reasonably acceptable, template-based docking with critical evaluation of the interfacial residue interactions could be considered a valuable tool to initially model protein interactions with other biomolecules, such as RNA. Therefore, evaluating or scoring these interfacial residue interactions should be carefully considered following the generation of this modeled complex (reviewed in [109]).

Apart from the challenges in modeling protein-RNA interactions, additional challenges remain in studying protein-RNA complex structures, including the fact that the interaction involves dynamics of biomolecules involved, both with respect to the RNA (which may also include modified RNAs), as well as with respect to the protein [107]. Nevertheless, the predicted complex structures from these computational modeling methods (such as template-based docking) can serve as a starting point for simulations to provide critical insights on modeled protein-RNA interactions with respect to refining such complexes, as well as to study their dynamics and provide an in-depth biophysical investigation of the complex with structural and energetic analysis [115], [116], [117], [118], [119], [120], [121].

### 4.3 Advances in computational methods for investigating protein interactions with modified RNA

Simulations have been widely employed to study protein-RNA interactions [122], [123]; advances in the development of force fields of RNA modifications [124], [125], and in the ability to parametrize chemical groups [126], [127] have laid the foundation for the computational study of the interface between proteins and the epitranscriptome using MD simulations. One such application of these advancements can be found in a high-throughput computational platform for screening protein targets for modified RNA recognition [128]. This protocol employed trees of chemical modifications to the four canonical nucleosides, with the complexity of the chemical modifications increasing along the branch points. Through short implicit solvent simulations, chemical modifications that led to favorable interactions with the protein of interest when compared to the simpler “parent” modification were selected and validated with longer, explicit solvent methods. This computational protocol was applied to the polynucleotide phosphorylase (PNPase) protein from *E. coli*, which has been previously investigated in human cells for its selective recognition of 8-oxoG [83]. Following the screening of the homology modeled PNPase structure, modifications predicted by the pipeline to show increased affinity with PNPase were tested *in vitro* alongside m^5^C, a modification screened out at the explicit solvent phase [128]. The experimentally determined binding affinities showed high correlation to the association free energy data from the explicit solvent MD simulations, showcasing the method’s ability to predict possible binding targets in a high-throughput manner. This synergistic experimental and computational approach, along with current techniques to generate the necessary protein and RNA structures, represents a starting point for further investigation of protein interactions with RNA modifications at an atomistic scale. Importantly, this platform served as a steppingstone for solving the “inverse problem” of examining the interaction of PNPase with 8-oxoG in atomic detail to provide insights into the mechanism of 8-oxoG discrimination [129]. Particularly, computations were employed to evolve PNPase for higher 8-oxoG affinity by screening mutants from a library of beneficial mutations and assessed their interactions using MD simulations [128]. Perhaps most importantly, improvements in 8-oxoG binding led to increased cell tolerance to oxidative stress, providing a clear link between molecular discrimination of RNA oxidation and cell survival. Overall, this methodology provided a framework for the rational engineering of modified RNA protein readers that could be applied to other systems outside of the studied PNPase.

## 5 Summary and outlook

The budding field of epitranscriptomics offers new and exciting opportunities for investigation of novel protein-RNA interactions. Large-scale studies to identify proteins that interact with a handful of RNA modifications have been conducted [28], [29], [30], [37], but relatively few of these proteins have been investigated on a mechanistic level. The current characterization of epitranscriptome reader proteins on this level is limited to proteins recognizing m^6^A, with the *in silico* investigations focusing on the YTH family of protein readers [59], [62], [64]. These studies demonstrate the power of atomistic simulations to reveal both the molecular basis for the YTH domain selectivity for m^6^A modified RNA and the possible redundancy in binding mechanisms for the YTHDF proteins. However, the YTH domain family represents only a single model of recognition for m^6^A, and the binding mechanisms for non-YTH domain readers such as the IGF2BPs [17] and HNRNPA2B1 [7], [77] require further investigation. Relatively few of the proteins identified have been investigated in terms of direct binding to the m^6^A modification [5], [7], [17], [76], [77] and fewer still for those proteins shown to interact with other modified RNAs such as m^1^A, m^5^C, and 8-oxoG. The *in silico* investigation of the YTH family of proteins represents a proof-of-concept for the power of atomistic MD simulations for mechanistic understanding of epitranscriptome reader proteins. The development of both protein structure prediction tools, molecular docking, and molecular mechanics parametrization of more than 100 different modified RNA species [124], [125] have offered support for conducting insightful synergistic computational and experimental studies into how these proteins recognize their modified RNA substrates. Further investigation of these intermolecular interactions holds great promise for uncovering new mechanisms of binding and molecular recognition of RNA modifications by proteins, leading to a rich understanding of how proteins recognize the epitranscriptome.

## CRediT authorship contribution statement

**Lucas G. Miller:** Conceptualization, Writing – original draft preparation, Reviewing & editing. **Madeline Demny:** Methodology, Writing – original draft preparation, Reviewing & editing, Visualization. **Phanourios Tamamis:** Conceptualization, Writing – reviewing & editing, Supervision, Funding acquisition. **Lydia M. Contreras:** Conceptualization, Writing – reviewing & editing, Supervision, Funding acquisition.

## Declaration of Competing Interest

The authors declare no conflicts of interest.
